# Chemical Composition, Antifungal Activity, and Plant-Protective Potential of *Rosa damascena* Mill. Essential Oil Against *Fusarium graminearum*

**DOI:** 10.3390/pathogens14040383

**Published:** 2025-04-15

**Authors:** Esma Özsoy, Timur Hakan Barak, Emre Yörük, Hüseyin Servi, Tapani Yli-Mattila

**Affiliations:** 1 Department of Molecular Biology and Genetics, Faculty of Sciences and Literature, Istanbul Yeni Yuzyil University, Cevizlibag, Istanbul 34010, Türkiye; esma.ozsoy@yeniyuzyil.edu.tr (E.Ö.); emre.yoruk@yeniyuzyil.edu.tr (E.Y.); 2 Department of Pharmacognosy, Faculty of Pharmacy, Acıbadem University, Ataşehir, Istanbul 34758, Türkiye; timur.barak@acibadem.edu.tr; 3 Department of Pharmacognosy, Faculty of Pharmacy, Istanbul Yeni Yuzyil University, Cevizlibag, Istanbul 34010, Türkiye; huseyin.servi@yeniyuzyil.edu.tr; 4 Department of Life Technologies/Molecular Plant Biology, University of Turku, FI-20520 Turku, Finland

**Keywords:** autophagy, *Fusarium graminearum*, real-time PCR, *Rosa damascena* essential oil, WST-1

## Abstract

*Fusarium graminearum* is a common plant pathogen among cereals worldwide. The application of chemical antifungal compounds is the most frequently used method in controlling *F. graminearum*. However, its excessive use and the genomic plasticity of the fungal genome lead to increased resistance levels to these chemical antifungal compounds. In this context, plant-derived compounds might play a role in protecting against Fusarium head blight (FHB) and crown rot (CR) as an alternative. In this study, we aimed to examine the antifungal effects of an essential oil obtained from *Rosa damascena* Mill. on the plant pathogen *F. graminearum* using molecular and analytical methods. The chemical composition of the essential oil was determined by GC-MS. The half effective concentration (EC_50_) value of *R*. *damascena* essential oil (REO) for *F. graminearum* was determined as 604.25 µg mL^−1^. Water-soluble tetrazolium 1 (WST-1) analyses revealed that REO caused cytotoxicity in *F. graminearum*. The potential oxidative stress and autophagic cell death capacity of REO towards *F. graminearum* was revealed via gene expression analysis and fluorescence microscopy. It was also revealed that, due to the plant-protective effect of REO, the disease severity of treated plants decreased by up to 27.78% in juvenile wheat seedlings infected by *F. graminearum*. Our data show that *R*. *damascena* essential oil might be used as an alternative natural ingredient in the field of plant protection.

## 1. Introduction

*Fusarium* species cause various diseases, including fusarium head blight (FHB) and crown rot (CR) in cereals, especially wheat and barley. *F. graminearum* and *F. culmorum* have been reported as the most common causal agents for FHB and CR worldwide [1,2,3,4]. *F. graminearum*, regarded as a species complex with more than 10 members, has been shown to be the predominant causal agent of FHB in many regions throughout the world. *F. graminearum* isolates lead to devastating effects, including yield losses, significant economic impacts, and mycotoxin contamination [5]. *F. graminearum* isolates produce various types of mycotoxins, such as zearalenone, nivalenol (NIV), deoxynivalenol (DON), and their derivatives, and these mycotoxins may lead to serious health problems in humans and animals [6,7,8,9,10]. Due to the relatively high incidence of *F. graminearum* and its mycotoxins in stored wheat and barley samples, it is very important to combat *Fusarium* species.

For many years, fungicides have been used against fungal pathogens as the main approach and disease management strategy. However, these chemical compounds have detrimental effects on the environment, animals, and human health [11,12]. In addition, due to the presence of increasingly aggressive fungal pathogens that are resistant to fungicides, there is a rising demand to limit the use of chemicals as antifungal agents in the field of disease management. Therefore, it is crucial to identify alternative antifungal compounds or strategies to combat FHB and CR diseases. These include biocontrol agents such as *Trichoderma* spp. [13,14,15] and the use of organic extracts from plants [16,17,18,19]. In particular, the potential for the application of antagonistic fungal treatment (such as *Clonostachys* spp., *Cladosporium* spp., and *Trichoderma* spp.) and essential oil mixtures such as *Combretum* spp. and *Ocimum* spp. in plant management appears promising for the near future.

According to previous research [17,20,21], essential oils, as plant-based materials, can play an important role in plant disease management. Numerous studies have demonstrated in recent years that essential oils demonstrate antifungal properties and can reduce the growth of fungal mycelia. Methanol extracts of *Combretum caffrum*, *Salix capensis,* and *Schotia latifolia* plantlets originating from South Africa have shown potential as antibacterial and antifungal agents for more than five pathogenic microorganisms. Similarly, *Cymbopogon citratus*, *Ocimum basilicum*, and *Ocimum gratissimum* essential oil treatment resulted in a reduction in both fungal growth and mycotoxin production in *Fusarium* spp. [21,22,23,24]. In addition, in comparison to fungicides, essential oils, as natural products, have various advantages in terms of sustainability, their modes of action, and environmental toxicity [25].

*Rosa* spp. are known for their beauty and healing properties. Rosaceae (rose family) is a rich plant family that includes at least 100 genera and more than 2000 species. Studies related to Rosaceae have mainly focused on its morphological classification, hybridization, anatomical analysis, and ecotype characterization [26,27]. However, the number of studies related to the processing, metabolite extraction, antioxidant characteristics, and antimicrobial potential of *Rosa* spp. has increased recently [28,29,30]. In this study, we aimed to determine the composition of *Rosa damascena* Mill. essential oil from Turkey via GC-MS and to show and characterize the potential antifungal effects against a worldwide phytopathogenic fungus, the *F. graminearum* species complex, in different physiological and molecular tests.

## 2. Materials and Methods

### 2.1. Plant Material

*R*. *damascena* buds (1 kg) were purchased from a local market in Türkiye. The plant materials were identified by Assoc. Prof. Huseyin Servi. The essential oil of *R*. *damascena* buds was obtained using a previously described method [31]. Briefly, 100 g *R*. *damascena* buds were soaked in 1000 mL distilled water and then extracted by hydrodistillation for 3 h using a Clevenger apparatus. Here, 1 kg *R*. *damascena* buds produced 1 mL essential oil.

### 2.2. Gas Chromatography/Mass Spectrometry Analysis

The essential oil was analyzed by GC-MS using a non-polar column, HP-5MS (5% phenyl, 95% methyl polysiloxane; 30 m × 0.25 mm, 0.25 m film thickness). The oven temperature was programmed as follows: isothermal at 60 °C for 1 min and then increased to 246 °C at a rate of 3 °C min^−1^ and subsequently kept isothermal for 30 min. The carrier gas was helium, with a flow rate of 0.9 mL min^−1^. The essential oil was injected (1 μL) in split mode. The identification of the compounds was performed by comparing the relative retention indices of the *n*-alkane series to the literature and with a mass spectrum comparison (NIST17 Mass Spectra Library). The relative percentage quantities of the separated compounds were calculated from the integration of the peaks in MS chromatograms [32].

### 2.3. Evaluation of Antifungal Activity

Dr. Tapani Yli-Mattila of Turku University provided the *F. graminearum* PH-1 reference strain. The *F. graminearum* PH-1 strain was incubated at 26 ± 2 °C in 9-cm-diameter Petri dishes on potato dextrose agar (PDA, Sigma Aldrich, St. Louis, MO, USA) and carboxymethyl cellulose (CMC) for 7 days. The experimental sets were grown on PDA medium amended with different concentrations of REO (0, 250, 500, 750, and 1000 µg mL^−1^; Sigma-Aldrich, USA). Meanwhile, 0.25 cm^2^ mycelial sample obtained from fresh PDA medium were used for cultivation. The MIC and EC_50_ values were calculated as described before [33]. For this purpose, the linear growth rate (mm/day) was calculated by measuring the radial growth length (mm) values at the 4th and 7th days of incubation. The half effective and maximum effective suppressor concentrations were determined as half effective (EC_50_) and minimum inhibitory concentration (MIC) sets, respectively. The final formula for EC_50_ calculation was as follows: EC50 = *x* + ((50 − y1)/(y2 − y1))*(*x* + *y*)/2, where *x* indicates a low inhibitor concentration (inhibition lower than 50%), *y* indicates a high inhibitor concentration (inhibition higher than 50%), y1 indicates the inhibition value for *x* concentration, and y2 indicates the inhibition value for *y* concentration.

### 2.4. Proliferation Analysis

The cytotoxic action of REO at the cellular level was determined by water-soluble tetrazolium 1 (WST-1; Roche, Switzerland) analysis. A 1 cm^2^ disc from the control and experimental groups was dissolved in 1X phosphate-buffered saline (PBS). Then, 10 µL (1:10 *V*:*V*) WST-1 (ScienCell, Carlsbad, CA, USA) was added to microplates containing 90 µL of the cells, and then the fungal cells were incubated for 3 h at 28 °C in the dark. Fold changes in cell proliferation were calculated by spectrophotometric measurement at 450 and 620 nm wavelengths [34].

### 2.5. Fluorescence Microscopy Assay

To determine the presence of oxidative stress and autophagic cell death responses in the tested strain treated with REO, 2′,7′-dichlorofluorescein (DCF-DA; Thermo, Waltham, MA, USA) and monodansylcadaverine (MDC; Sigma Aldrich, USA) staining tests were carried out via fluorescence microscopy (Carl-Zeiss, Baden-Württemberg, Germany). Mycelial plugs obtained from 7-day-old PDA cultures were transferred to CMC medium. Then, liquid cultures were incubated on a rotary shaker for 120 rpm at 26 ± 2 °C. The 7-day-old liquid cultures were filtrated with 2X sterile gauze to remove the mycelium. The obtained 1 × 10^5^ macroconidium cultures were used in fluorescence microscopy analysis. Fixation, stain treatment, washing, and cell collection steps were carried out following previous procedures [20,35,36]. Quantitative assays were carried out using 100 cells per replicate (*n* = 3).

### 2.6. Real-Time Polymerase Chain Reaction (qRT-PCR) Assays

To analyze the changes in gene expression, qRT-PCR assays were conducted. For this purpose, total RNA was isolated from fungal samples via Triagent (Macherey-Nagel, Düren, Valencienner Str., Germany). The manufacturer’s recommendations were followed for the binding, washing, and eluting processes. After DNase I treatment (Zymo, Irvine, CA, USA), spectrophotometric (260/280 nm absorbance) measurements and agarose gel (0.8%) assays were used in order to reveal the quantity and quality of the total RNA. Then, the total RNA was converted to cDNA molecules via a commercial kit (Nepenthe, TürkiyeTurkey-Kocaeli), and the cDNA molecules were used in gene expression analysis. In the qRT-PCR analysis, β-tubulin (AY303689) was used as an endogenous gene. The autophagy protein 5 (atg5, FGRA07_10212) and catalase_3 (cat, FGSG_06733) genes were used as target genes, and they were associated with different physiological processes, i.e., the autophagy response and oxidative stress. Mitogen-activated protein kinase (mgv1, AF492766.1), related to sexual–asexual growth, was used as a positive calibration gene in order to identify potential alterations in gene expression. The primers used in this study are given in Table 1. The qRT-PCR assays were conducted with mixture including a volume of cDNA corresponding to 50 ng total RNA, 3 pmol of each primer, and 1X SYBR Green I Master Mix (Episozyme, Turkey), with the remaining volume consisting of nuclease-free water. The cycling conditions were as follows: 95 °C for 2 min (pre-denaturation); a loop with 40 repeats consisting of 95 °C for 10 s, 58 °C for 15 s, and 72 °C for 20 s; 40 °C for 30 s (cooling); and a melting step (Applied Biosystems, England). Fold changes in the gene expression values were calculated using the 2^-ΔΔCT^ formula, which is presented as follows: 2^−ΔΔCT^ = 2^−[ΔCT sample − ΔCT control]^, where ΔCT is CT_target_ − CT_reference gene_ [37]. Each experiment was replicated with at least three biological replicates and two technical replicates.

### 2.7. Infection Tests for Fusarium Crown Rot (FCR) Disease

*Triticum aestivum* L. cv. Lucilla seeds (kindly provided by the Seed Progen Company, Turkey) were used in plant protection tests for juvenile plantlets that were infected with the *F. graminearum* PH-1 strain. The seeds were firstly surface-sterilized with 0.64% NaOCl for 5 min, 10% ethanol for 5 min, and sterile dH_2_O at least three times. The seeds were transferred to filter papers inside Petri dishes; they were incubated at 26 °C (in the dark) for 3 days. CMC cultures were used as the inoculum source. Then, 1 × 10^6^ macroconidia were filtered through 2X gauze, and then they were added to autoclaved soil at a ratio of 5:95/*W*:*V* macroconidia–soil. The wheat seeds [38,39,40] were planted in 13-cm-diameter plastic pots containing soil with *F. graminearum* and incubated at 26 °C in a 16/8 h light/dark cycle with 1500 lux for two weeks. Plantlets were watered with distilled water (control set) or an REO solution. Juvenile plantlets were evaluated regarding the FCR disease scale, ranging from “0” to “4”, with three biological replicates each, including three technical replicates [41,42]. The disease severity as a percentage of infection was calculated according to the formula disease severity (percentage) = 100 × (Σ (n × V))/(Z × N), where “n” is the number of samples with different scores, “V” is the value for the scale, “Z” is the highest value of the sample scale, and “N” is the sample number.

### 2.8. Statistical Analysis

The column statistics, including the mean and standard error values, as well as normality tests and *t*-tests, were obtained using the software GraphPad Prism, version 9.0 (Dotmatics, Boston, MA, USA). The correlation matrix, using Pearson’s coefficients and principal component analysis (PCA), was constructed using the R programming language. The “Hmisc”, “corrplot”, “ggcorrplot”, “PerformanceAnalytics”, and “ggbiplot” packages were used in the correlation matrix and PCA graphics formation processes. The R terminal commands have been deposited in the GitHub repository (please see https://www.github.com/eyoruk, (accessed on 8 September 2024)). The confidence interval was 0.05. Each experiment was carried out with three biological replicates and two technical repeats.

## 3. Results

### 3.1. Phytochemical Evaluation

**Figure 1:**
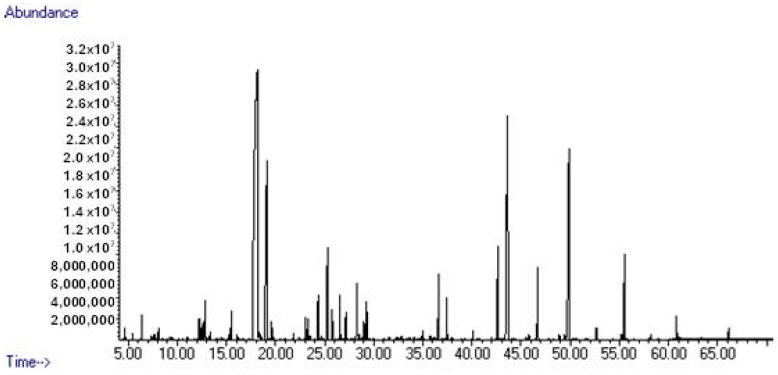
GC-MS chromatogram of *R*. *damascena* essential oil.

*Rosa damascena* is highly prominent due to its essential oil content, which has significant economic and biological value. Thus, numerous studies have been conducted to reveal its phytochemical profile. Gas chromatography with tandem mass spectrometry (GC-MS) is usually considered as the standard and most convenient method for the examination of phytochemical ingredients in essential oils. For these reasons, GC-MS was used to reveal the phytochemistry of REO in this study. Forty-five compounds were identified, constituting 99.6% of the REO; see Table 2 and Figure 1. Citronellol (37.1%), nonadecane (13.8%), geraniol (10.6%), and heneicosane (8.9%) were observed as the main compounds of the essential oil, and monoterpenoids (52.3%) and *n*-alkane derivatives (35.3%) were identified as the dominant groups.

### 3.2. Antifungal Treatment Analysis

The radial growth rate based on EC_50_ determination was determined using a common agar dilution technique. *F. graminearum* PH-1 cultures were grown on PDA medium amended with REO, including 0, 250, 500, 750, and 1000 µg mL^−1^ REO. While the 1000 µg mL^−1^ REO-amended PDA medium suppressed fungal growth entirely, the remaining PDA media with different concentrations of REO showed only the partial suppression of fungal growth. According to the radial growth formula, the EC_50_ value was calculated as 604.25 µg mL^−1^ REO treatment. Further studies, including transcriptional, epigenetics, physiological, and phytoprotection tests, were carried out with the control and EC_50_ sets.

The WST-1 assay was used in order to reveal the potential cytotoxic effects of REO on the *F. graminearum* PH-1 strain. Similarly, the relative inhibition of in vitro growth was detected in the WST-1 analysis. The relative absorbance values (Δ450–620) for the control and experimental sets were calculated as 0.18 ± 0.0 and 0.1 ± 0.0, respectively. The fold change in cell proliferation was recorded as 58.88 ± 3.53%, which corresponded to a significant change with *p* < 0.001.

### 3.3. Fluorescence Microscopy Analysis

**Figure 2:**
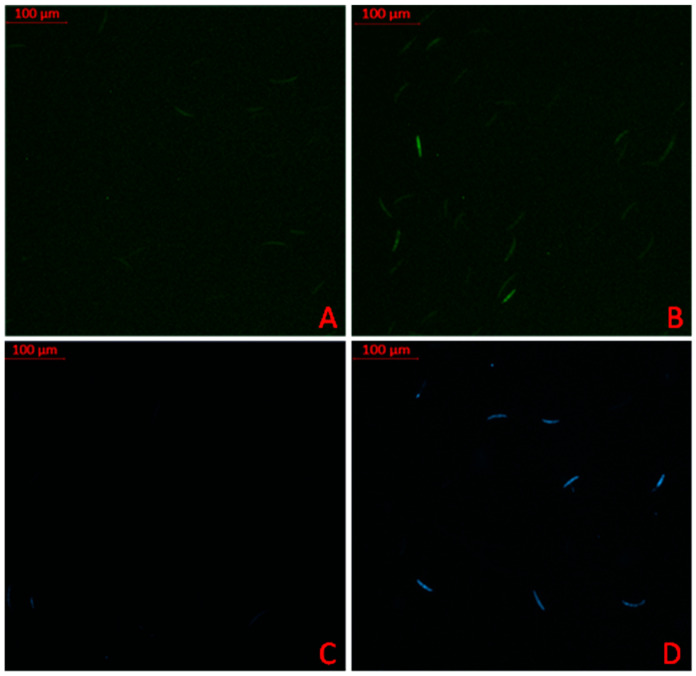
Fluorescence profiling of REO-treated *F. graminearum* PH-1 strain for DCF-DA (**A**,**B**) and MDC (**C**,**D**) tests. (**A**,**C**) illustrates control sets for DFC-DA and MDC treatment, respectively. Similarly, (**B**,**D**) illustrate REO sets for DCF-DA and MDC treatment.

The potential oxidative stress and autophagic cell death induction effects of REO on the *F. graminearum* PH-1 strain were evaluated via DCF-DA and MDC staining, respectively. The staining profile for 100 cells per biological replicate was determined. The DCF-DA staining percentages for the control and experiment sets were recorded as 20.33 ± 6.06 and 31.67 ± 5.23, respectively (*p* < 0.05). A similar increase with a significant difference (*p* < 0.05) in the stained cell percentage was observed in the control (25.67 ± 3.180) and experiment (39.00 ± 4.04) cells in the MDC staining tests. Figure 2 shows cells stained with oxidative stress and autophagy fluorescence agents.

### 3.4. Gene Expression Analysis

**Figure 3:**
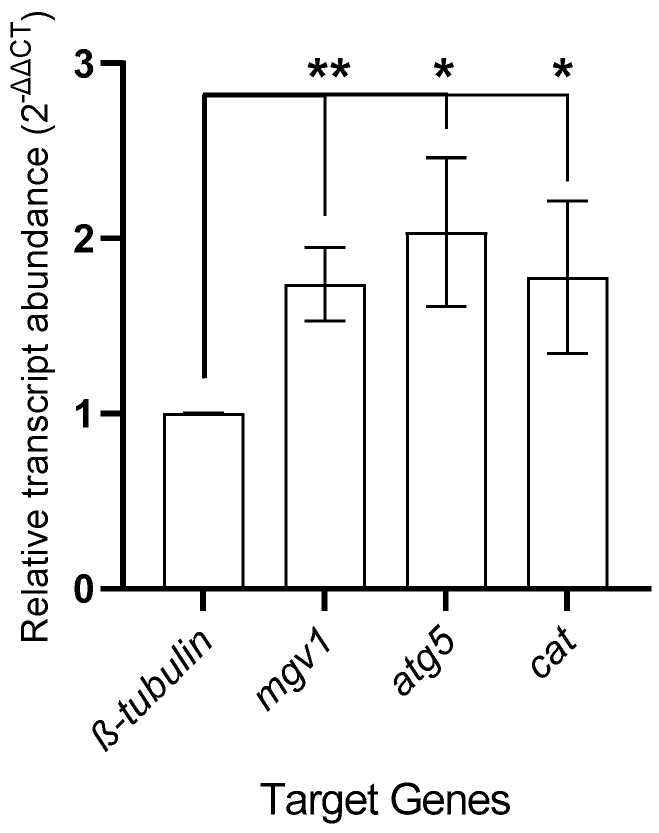
Fold changes in expression of genes related to autophagy, oxidative stress, and sexual–asexual reproduction in *F. graminearum* PH-1 strain. Significant changes were yielded with different levels of alteration, where “*” means *p* < 0.05, ** means *p* < 0.01.

The β-tubulin normalized gene expression analysis included fold changes in genes related to autophagy (atg5: autophagy protein 5), oxidative stress (cat: catalase_3), and sexual–asexual growth kinetics (mgv1: mitogen-activated protein kinase). The 2^−ΔΔCT^ value for the atg5 gene was calculated as 2.03 ± 0.42 (*p* < 0.05). Similarly, upregulation in the REO-treated *F. graminearum* PH-1 strain was detected for the cat gene. The fold change in the cat gene was recorded as 1.77 ± 0.43 (*p* < 0.05). Sexual and asexual growth-related mgv1 expression was used to validate the potential abiotic and/or biotic stress factor presence via qRT-PCR analysis. This revealed similarly upregulated expression for the mgv1 gene with 2^−ΔΔCT^ values of 1.73 ± 0.21 (*p* < 0.01) in the REO-treated *F. graminearum* PH-1 strain. Figure 3 shows the alterations in gene expression in the REO-treated *F. graminearum* PH-1 strain.

### 3.5. Disease Severity Analysis

**Figure 4:**
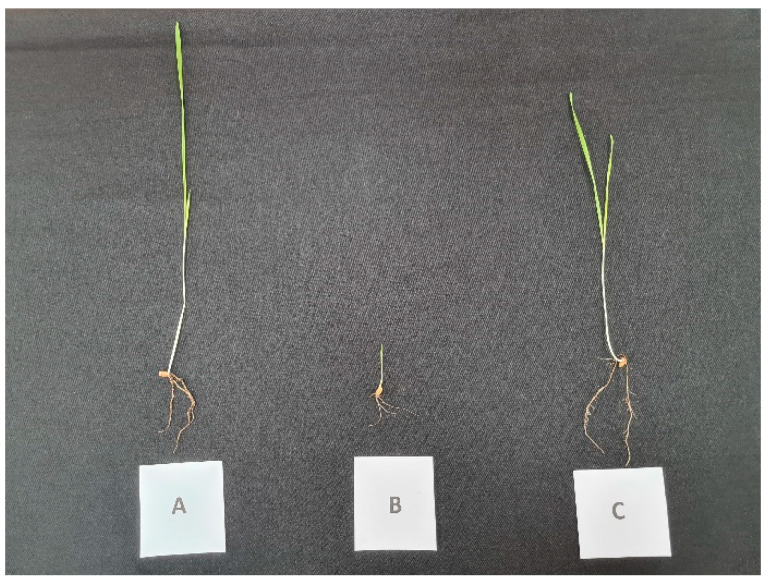
(A) Control, (B) *F. graminearum* PH-1-infected, and (C) REO-treated infection sets of juvenile plantlets of *T. aestivum* L. cv. Lucilla.

*F. graminearum* PH-1 infection led to a significant level of FCR disease severity in *T. aestivum* L. cv. Lucilla, a wheat cultivar with high yield potential. The disease scores ranged from one to three. The FCR disease severity was calculated as 80.56 ± 5.31%. REO-treated juvenile plants showed similar shoot and root development profiles in wheat. The disease severity was calculated as 52.78 ± 11.45. Significant changes were found in the regression of the disease symptoms (*p* < 0.05). Figure 4 shows plantlets belonging to the control, infected, and REO-treated infected sets.

### 3.6. Statistical Analysis

**Figure 5:**
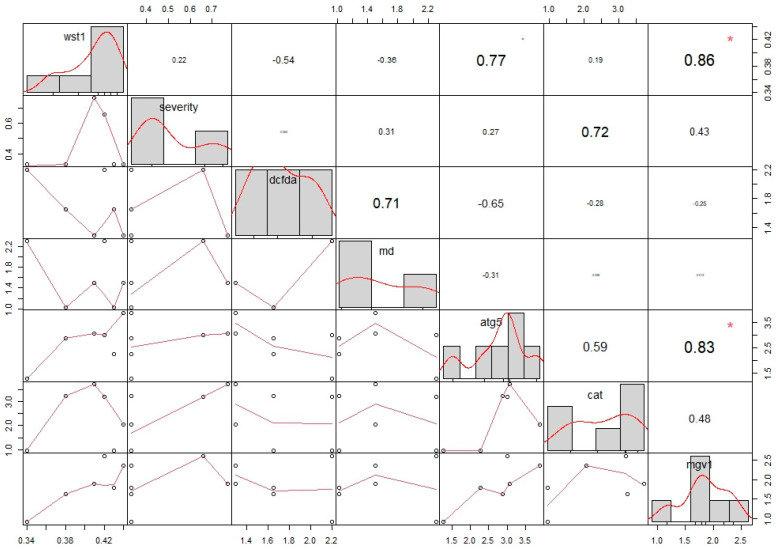
The correlation matrix obtained with Pearson’s coefficients, showing a highly heterogeneous distribution and fold changes in different data sets. “*” means *p* < 0.05.

**Figure 6:**
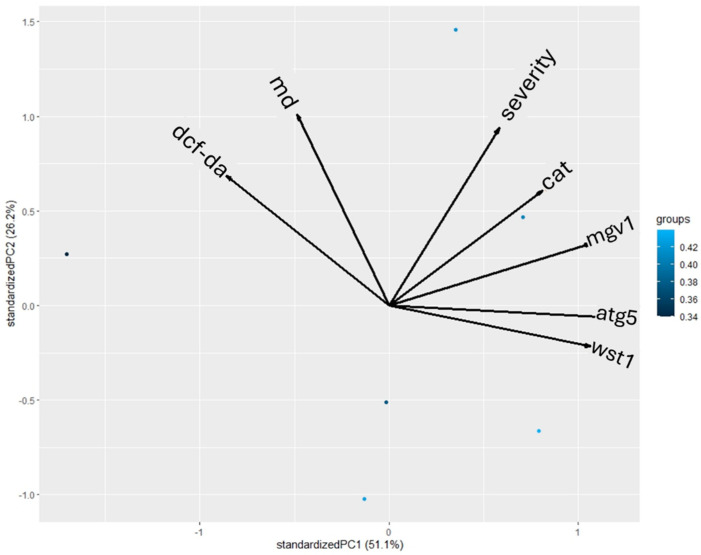
PCA profiling of different data sets obtained in this study. “atg5”, “mgv1”, and “cat” denote gene expression data in *F. graminearum* PH-1, while “dcf-da” and “md” denote fluorescence microscopy data (oxidative stress and autophagy test) and “wst1” refers to the cell proliferation test. Finally, “severity” refers to the FCR disease assessment in *T. aestivum* L. cv. Lucilla.

The correlation matrix (CM) was supported by Pearson’s correlation coefficients and principal component analysis (PCA). Each value was normalized to a fold change of “1”. The CM revealed that only a positive correlation was present in the limited data sets. No negative correlation was present in the data sets with significant differences (*p* > 0.05). The WST-1 assay and mgv1 expression showed similar patterns of upregulation (Table 3). These two data sets showed a positive correlation, with a *p* value = 0.02 and r value = 0.86. Similarly, a positive correlation was found between mgv1 and atg5 expression. The *p* and r values were calculated as 0.04 and 0.83, respectively (Figure 5). A highly heterogenous distribution among the different data sets was observed through the correlation matrix (Figure 5). Similarly heterogeneous profiling was indicated by the PCA tests. The percentage for the first and largest component was calculated as 51.1%. The second-largest percentage was 26.2% (Figure 6).

## 4. Discussion

It is apparent that fungicide treatment still dominates fungal disease management in crops worldwide. The list provided by the Fungicide Resistance Action Committee (FRAC) provides comprehensive data regarding the characteristics of fungicides used for disease management worldwide, being updated yearly [43,44], and the latest version of the list is provided on the FRAC Code List website (please see https://www.frac.info/ (accessed on 15 January 2025)). The fungicides belonging to the classes C2, C3, and G have been adapted to be used on crop fields. However, resistance development, the usage of fungicides with unknown modes of action (class U), and some other ecological issues could cause problems in the near future [45,46,47,48]. Therefore, it is necessary to incorporate alternative practices into the control strategies for plant diseases.

It can be observed that plant-derived secondary metabolite application is a major topic, offering an alternative to the use of fungicides [49]. In particular, mixtures including essential oils or specific plant metabolites such as camphor and thymol have been shown to be in vitro growth suppressors for *Fusarium* spp. However, the majority of existing studies include only the analytical compositions of these plant-derived secondary metabolites and MIC and sub-MIC determination assays [50,51,52,53]. The mechanistic routes behind the potential antifungal effects of these mixtures or specific compounds are still missing from the literature. Here, we aimed to reveal the chemical composition of *R*. *damascena* essential oil, to present its potential antifungal effects on the worldwide phytopathogenic fungus *F. graminearum* PH-1, to determine the phytoprotective effects of REO on wheat, and to show the destructive effects of REO on *F. graminearum* via different methods.

It is known that the ingredients of essential oils can be highly diverse due to various parameters, including the climate, geography, processing, etc. [54]. In a study, *Rosa damascena* samples were collected from four different regions of Iran, and a GC-MS analysis was conducted on the essential oils. The results showed that citronellol was the major ingredient for all samples; however, the amount varied between 22.4 and 35.2% [55]. In another study, the effect of the harvesting time on the chemical profile of REO was investigated. The results demonstrated that geraniol and citronellol were the dominant ingredients; however, most major ingredients were altered due to the harvesting time [56]. Similar results were observed in a study conducted in Turkey. The harvesting time of *Rosa damascena* samples from Mardin province significantly altered the chemical composition [57]. In another study, researchers obtained essential oils from samples harvested at the same province in Turkey. The phytochemical profile obtained was highly comparable to that observed in our work. Citronellol was detected as the major ingredient; in addition, geraniol, nonadecane, and heneicosane were observed in significant amounts [58]. These results show that the phytochemical profile of the REO sample used in this study is analogous to those described in the literature.

Fortunately, knowledge regarding the potential antimicrobial effects of REO has been increasing. However, there are few studies concerning the compound composition and the antifungal effects of REO on *F. graminearum*, as well as its plant-protective effects. In contrast, studies investigating the antifungal effects of REO are increasing. In a previous study, REO from Iran was investigated against various bacterial and fungal strains in waste and well water [59]. The results showed that REO significantly inhibited *Candida albicans* strains, with an MIC = 62.50 μg mL^−1^. In another study, five different samples from Saudi Arabia were investigated for their inhibitory effects against various bacterial strains and a fungal strain, *Aspergillus flavus*. The results showed that all samples significantly inhibited fungal development [60]. Another study investigated REO against *Aspergillus flavus* along with 74 other essential oil samples. The results demonstrated that REO was one of the most active essential oils among 75 samples [61]. The volatile organic compounds from *Rosa damascena* were studied in a prior report against fungal strains of *Fusarium oxysporum* and *Fusarium graminearum*. The results demonstrated that VOCs from *R. damascena* showed the greater inhibition of *F. graminearum* [62]. Although there are a few reports of REO’s activity against fungal strains, there is a scarcity of studies investigating the antifungal properties of REO in detail from a mechanistic point of view. For these reasons, in this study, the antifungal and plant-protective properties of REO were investigated comprehensively.

In a previous study, 3-(4,5-dimethylthiazol-2-yl)-2,5-diphenyltetrazolium bromide (MTT) assays on *Candida albicans* showed that REO significantly suppressed fungal growth, similarly to the data from the WST-1 assay in this study. A relatively lower MIC value was found in the determination of the phytoprotective effects of REO on *F. graminearum*-infected wheat plantlets. Our results showed that REO treatment positively reduced the adverse effects of *F. graminearum* infections in wheat. The findings obtained from this study reveal that REO required a relatively low concentration for treatment in in vitro and semi-in vivo antifungal tests.

The potential oxidative stress- and autophagy-mediated antifungal effects of REO on *F. graminearum* PH-1 were tested by gene expression analysis and fluorescence microscopy tests. There are only limited investigations of the potential oxidative stress- and autophagy-related effects of REO on fungal species. The cell membrane integrity, mode of action, and antioxidant assays using *C. albicans* and *Fusarium* spp. have been reported previously. The results showed that the SYTOX-green and DCF-DA staining protocols are effective tools in conducting oxidative stress and mode of action tests for REO [63,64]. Gene expression studies on the oxidative stress and autophagic cell death effects of REO on *F. graminearum* are still missing from the literature. Our results showed that REO led to increased oxidative stress levels in REO-treated fungi, and autophagic cell death was present in the REO-treated *F. graminearum* PH-1 strain. While there was no positive correlation between the gene expression and fluorescence microscopy results, both test types revealed significant alterations due to REO treatment in *F. graminearum*. Interestingly, PCA profiling showed that the fluorescence microscopy and gene expression data were clustered separately. While the DCF-DA and MDC tests were closely related on the second axis, the gene expression tests were distributed on the first axis. However, both the correlation matrix and PCA profiling assays revealed that mgv1 expression, which is directly related to asexual and sexual development, showed a positive correlation with and a similar distribution pattern to atg5 expression. The significant level of correlation between vegetative reproduction and autophagy processes could indicate the destructive presence of stress within a fungal cell. However, further studies aimed at providing detailed information about the antifungal effects of REO are needed. In particular, studies including the investigation of the crosstalk between autophagy and other programmed cell death types, including apoptosis and necroptosis, would provide data about the interconnected and complementary pathways behind the potential antifungal effects of REO.

## Figures and Tables

**Table 1:** qPCR primers used in this study.

Gene	Primer Set	Forward Sequence/Reverse Sequence (5′–3′)	Band Size (bp)
β-tubulin	betaF/betaR	AGGGTCATTACACCGAGGGT/GTACCACCACCAAGAGAGTGG	121
FgMgv1	MgvRTF/MgvRTR	AGGTTCAACGATTCCGACAG/ GACCATTACCCTGAGGCAGA	100
catalase_3	CatrtF/CatrtR	AATTCCACGTTCGTTTCGTC/ CCATACTAGGCTCGCTTTGC	130
atg5	Atg5rtF/Atg5rtR	ATGTCTTCTCCCATCCCGC/ GCTGAAGCGTGGAATACTGG	108

**Table 2:** Chemical composition of *R*. *damascena* essential oil.

RT ^1^	RRI Exp. ^2^	RRI Lit. ^3^	Compound	(%)
5.438	903	899	Heptanal	0.1
6.351	934	939	α-Pinene	0.4
7.374	969	970	1-Heptanol	0.1
7.647	978	980	β-Pinene	0.1
8.070	992	991	Myrcene	0.2
12.223	1103	1098	Linalool	0.5
12.351	1106	1102	Nonanal	0.3
12.636	1113	1111	*Cis*-Rose oxide	0.4
12.810	1117	1117	Phenylethyl alcohol	1.1
13.318	1129	1126	*Trans*-Rose oxide	0.2
15.227	1174	1172	Nonanol	0.2
15.483	1180	1177	Terpinen 4-ol	0.8
16.090	1195	1189	α-Terpineol	0.1
18.149	1243	1228	Citronellol	37.1
18.212	1244	1237	Isogeraniol	0.1
18.332	1247	1240	β-Citral	0.3
19.118	1265	1255	Geraniol	10.6
19.585	1276	1270	α-Citral	0.5
21.801	1328	1323	(*E*)-Methylgeranate	0.1
23.036	1357	1354	Citronellol acetate	0.5
23.268	1363	1356	Eugenol	0.6
24.328	1388	1365	Neryl acetate	1.1
25.273	1411	1401	Methyl eugenol	2.7
25.761	1423	1418	(*E*)-Caryophyllene	0.8
26.544	1442	1443	α-Guaiene	1.2
26.672	1445	1449	β-Phenylethyl butyrate	0.1
27.157	1457	1455	α-Caryophyllene	0.8
28.285	1485	1480	Germacrene D	1.6
28.482	1490	1485	β-Selinene	0.2
28.995	1502	1500	Pentadecane	0.5
29.264	1509	1495	δ-Guaiene	1.0
36.590	1703	1700	Heptadecane	1.8
37.429	1727	1717	*Trans*-Farnesol	1.2
40.084	1803	1800	Octadecane	0.2
42.615	1878		9-Nonadecene	2.9
43.635	1909	1900	Nonadecane	13.8
45.801	1977		1-Eicosene	0.1
46.685	2005	2000	Eicosane	2.1
49.398	2093		Henicos-1-ene	0.2
49.852	2108	2100	Heneicosane	8.9
52.662	2204	2200	Docosane	0.3
55.222	2295		1-Tricosene	0.1
55.520	2306	2300	Tricosane	2.6
60.807	2505	2500	Pentacosane	0.7
66.127	2705	2700	Heptacosane	0.4
			Monoterpenes	0.7
			Monoterpenoids	52.3
			Sesquiterpenes	5.6
			Sesquiterpenoids	1.2
			*n*-Alkane derivatives	35.3
			Others	4.5
			Total	99.6

^1^ RT: retention time; ^2^ RRI Exp.: relative retention index calculated against *n*-alkanes (C5-C30); ^3^ RRI Lit: relative retention index given in the literature for the compound in similar columns and analysis conditions.

**Table 3:** Pearson’s r correlation matrix obtained with separate data sets, including those for the *F. graminearum* PH-1 strain (WST-1 toxicity test (wst1), DCF-DA oxidative stress test (dcfda), MDC autophagy test (md), gene expression assays (atg5, cat, and mgv1)), and *T. aestivum* L. cv. Lucilla (FCR severity test (severity)).

r correlation values obtained via Pearson’s correlation matrix							
	wst1	severity	dcfda	md	atg5	cat	mgv1
wst1	1.00	0.22	−0.54	−0.36	0.77	0.19	0.86
severity	0.22	1.00	−0.06	0.31	0.27	0.72	0.43
dcfda	−0.54	−0.06	1.00	0.71	−0.65	−0.28	−0.25
md	−0.36	0.31	0.71	1.00	−0.31	−0.06	−0.01
atg5	0.77	0.27	−0.65	−0.31	1.00	0.59	0.83
cat	0.19	0.72	−0.28	−0.06	0.59	1.00	0.48
mgv1	0.86	0.43	−0.25	−0.01	0.83	0.48	1.00
*p* values obtained via Pearson’s correlation matrix							
	wst1	severity	dcfda	md	atg5	cat	mgv1
wst1		0.66	0.26	0.47	0.07	0.71	0.02
severity	0.66		0.90	0.55	0.60	0.10	0.39
dcfda	0.26	0.90		0.11	0.15	0.59	0.63
md	0.47	0.55	0.11		0.54	0.91	0.98
atg5	0.07	0.60	0.15	0.54		0.22	0.04
cat	0.71	0.10	0.59	0.91	0.22		0.33
mgv1	0.02	0.39	0.63	0.98	0.04	0.33	

## Data Availability

The original contributions presented in this study are included in the article. Further inquiries can be directed to the corresponding author.
